# A Case of Atresia Vaginæ in a Parturient Female

**Published:** 1857-01

**Authors:** 


*A Case of Atresia Vaginæ in a Parturient Female.* By M. PENDLETON, M. D., Louisa County, Va. Operation of Vaginal Section. Delivery with Forceps. By J. H. CONWAY, M. D., Professor of Obstetrics, &c., in Medical College of Virginia.

### *Previous History of the Patient, by Dr. Pendleton.*

Mrs. * * * of the county of Louisa, fifty miles from Richmond, aged twenty-four years; married seven years; always enjoying a vigorous constitution; had what was supposed to be an abortion in the spring of 1853, the process being protracted, and attended with a most alarming flooding. The physicians in attendance very properly used the tampon, and from the obstinacy of the hæmorrage, and the extreme exhaustion of the patient, felt constrained to let it remain for some days. Being called in the meantime in consultation with the attending physicians, I found her as pale as death, her pulse countless, and scarcely perceptible, and the vital forces so nearly exhausted, that no concern was felt by any of us about the tampon that still remained in the vagina, whilst every attention was given to such means as promised to keep soul and body together. At length, the symptoms improving, the tampon was removed. The patient continued in a most exsanguined condition for some time, but gradually improved, until her health seemed fully restored in twelve months from this attack.

About this time she suffered excruciating pain in the uterus, which lasted for several days, and then gradually subsided, leaving the patient with an uncomfortable sensation of dull pain and weight about the womb. She continued to experience these paroxysms once a month for some time, but never menstruated.

I was again consulted, and so great were her sufferings that we gave her chloroform. The next morning there was a sudden gush of the most offensive secretions, which we took to be long retained menstrual fluid. After this her health improved steadily, the menses flowing at the regular periods—sometimes with difficulty and attended with pain, and sometimes with no pain or difficulty whatever.

Her family physician, Dr. Isbell, now no more, told me that he made a speculum examination, and that he found a perfect occlusion of the vagina, yet she continued to menstruate, as above stated, for months, until about the last of November, 1855, when she exhibited signs of pregnancy; from which period until parturition commenced, she enjoyed the most perfect and blooming health.

When taken with labor, I was called to attend her. Her pains were strong but short, and at intervals of five to ten minutes. Remembering the history of her case, with no little solicitude I proceeded to make a digital examination. At once it was too evident that there was atresia of the vagina, about four inches from the external vulva. There were several slightly projecting teats—feeling like warts or projecting cicatrices, the largest of the size of a large pea—the others much smaller. Not the least opening could be detected by the finger. These teats were on the left side of the septum or partition formed by the closed vagina. In the absence of uterine contractions, something firm like the head of the child was felt, but so thick was the dividing wall that it was difficult to say certainly what was the prominent presentation. During the contractions of the uterus nothing firm could be felt; but on the contrary, there was an elastic and somewhat fluctuating sensation; which signs became more and more distinct as labor continued; and the partition wall having grown thinner, no doubt was left that the head presented, and that the waters were protruded and were pressing the septum forward with a violence which made me fear that the whole vagina would be torn from its attachments. Finding that things were at an awful pass, and that nothing short of an operation could save the mother from certain death, and viewing the case as one of great complexity and danger, I had, very early in the affair, called in a promising young physician, who had but recently settled in the neighborhood, (Dr. John Gardner,) who fully concurred with me in every particular. We at once determined to send to Richmond for Dr. Conway, the distinguished professor of obstetrics in the Virginia Medical College, who, with Dr. Peachy, arrived about half past 10

o'clock, A. M., on the 28th of August ultimo, about thirty-six hours from the commencement of labor.

I will not describe the case more fully, as I only designed to give the previous history, preferring that my friends Drs. Conway and Peachy should describe the operation they so skillfully and successfully performed.

I will only add that under the most rigid antiphlogistic treatment, there followed but little inflammation and fever, and adhesion was prevented by passing the finger once a day in a circular direction where the septum had been. Injections of milk and water (sometimes flax seed tea,) were thrown up, with as much force as possible, once a day—and now, I am happy to say, both mother and child are well.

### *History of the Operation, by Dr. Conway.*

On the morning of the 28th of August last, I received a note from Drs. Pendleton and Gardner, requesting me to visit Mrs. W., of Louisa county. They stated that she was in labor, and had occlusion of the vagina. I immediately made preparation to go, and with my friend Dr. Peachy, who kindly consented to accompany me, reached the residence of Mr. W. about 11 o'clock, A. M.

After hearing the history of the case, I proceeded to make a vaginal examination by the touch, and found a membranous septum extending from a point a few lines above the meatus urinarius upwards and backwards towards the posterior cul-de-sac of the vagina—thus dividing that canal into two cavities, an inferior or vulvar, and a superior or uterine cavity. The anterior origin of the atresia was about an inch from the vulva, while the posterior attachment was distant from the commissure of the perineum about two and a half inches—making the posterior wall of the vulvar cavity one and a half inch longer than the anterior.

I also found, as Dr. Pendleton informed me I would, four or five carunculæ about one-fourth of an inch behind the mouth of the urethra, in the centre of which I felt a depression that I thought possibly might be the meatus itself, drawn backwards by the adhesion between the anterior and posterior walls of the vagina. During a pain I could distinctly feel a bulging of the parts, and to the touch they conveyed a sense of fluctuation, as if the os uteri were fully dilated, and the tumor formed was caused by the descent of the membranes, but it was caused for the most part by an accumulation of "the show." During the intervals of the pains I could distinguish a hard, resisting surface, which I took to be the head of the child.

Drs. Pendleton and Gardner having already explored the parts, I requested Dr. Peachy to make an examination; which he did. Upon returning to another room, in conference, we determined of course that before we could conclude upon the proper mode of conducting the case, it would be necessary to make an occular inspection of the vagina.

I accordingly placed the lady in a proper position before a window, and first by separating the labia, and by the speculum, explored the parts thoroughly. Near the centre of the *field of the speculum* I found the little mass of the carunculæ, and by the introduction of an ordinary silver probe, which met with some resistance, I found an orifice. I then succeeded in gradually introducing an olive pointed gum-elastic catheter, which was carried above the septum as far as the length of the instrument would permit. I then withdrew the speculum, and passed another catherer into the urethra, leaving the first in situ. Both instruments were pushed in to their full length, and when withdrawn were covered with "the show," manifesting, as no urine passed through the urethral catherer, that there was an urethro-vaginal fistula, and that both instruments had entered the same cavity. Being still doubtful about the thickness of the septum, and ignorant of the condition of the parts above it and beyond our sight, we concluded that we had recourse to but two modes of effecting delivery, either by a vaginal section or division of the septum, or, *as a dernier resort*, the Cæsarian operation.

As Mrs. W 's condition was favorable, though she had been in labor for thirty-six hours, we determined to wait for a time, with the hope that natural delivery would take place. I was led to this determination by my recollection of a case somewhat similar, that occurred in the infirmary of the Medical college of Virginia, under the charge of the late Prof. Bohannan, who thought that possibly it might be necessary to perform the Cæsarian section, but determined to delay until he saw what the natural forces would accomplish. After a short time, in that case, the head descended upon the adhesion, which gradually separated, and the termination was favorable to both mother and child.

After a delay of two or three hours, finding that no salutary progress had been made, and, as especially shown by her countenance, that symptoms of exhaustion would soon supervene, we determined that it would be best to make an exploratory incision into the the septum, and then, if no contra-indication existed, to extend the incision so as to convert the two cavities into a continuous canal.

After placing the patient in a state of anæsthesia, I put her in the position for the operation of version, and sat in a chair in front of her and between her limbs, and whilst Dr. Peachy separated the vulva, I proceeded to operate. I introduced the index finger of my left hand to the point of the opening, and with a straight probe-pointed bistoury, made a slight incision downwards and backwards towards the rectum.

I am aware that we are advised against making this incision in such cases, lest we might infringe upon the rectum; but the depth of the septum from the point of the incision to the rectum was so great as not to render the cut hazardous. Besides, that portion of the septum at the orifice and surrounding it, was of a dense, fibrous structure, and therefore unyielding, while the rest was soft and fleshy, partaking the character of the original tissue.

Hence, had the labor progressed further, the probability is that dilation would not have taken place, but the septum would have been lacerated at its periphery instead of the orifice, and thus injury to the rectum and urethra would have occurred.

I now introduced the index finger of my left hand, and pressing upwards for the purpose of shielding the urethra, and passing the same knife along it, made two horizontal incisions—one to the left and another to the right—and then, by dint of tearing and occasionally cutting in the three directions indicated, I succeeded in removing almost the whole obstruction.

I found, upon passing my finger above the point where the atresia had existed, that the os uteri was fully dilated, the bag of waters formed, and diagnosticated the left occipito anterior iliac position of the vertex. Having removed what we conceived to be the whole difficulty, after rupturing the membranes, we determined to leave the case to nature to complete her work, except that, finding the pains were growing feeble, we administered two or three doses of ergot, which accelerated the pains.

In a short time the head descended into the cavity of the pelvis as far as the point of the adhesion, where it remained without much if any advancement for about three hours.

During the whole period the fœtal circulation could be distinctly heard; and after delaying for the three hours, although we thought it probable that nature would finally accomplish the delivery, yet we deemed it ill advised to permit parts, which had been already subjected to violence by the previous operation, to undergo longer continued pressure, and in order certainly to preesrve the child, agreed that the time had arrived when it was proper to resort to artificial delivery, and determined to apply the forceps.

I accordingly again brought the patient to the edge of the bed, and taking the position I had previously assumed, requested Dr. Peachy to prepare and hand me the instruments, and at the proper time to support the perineum. I applied first the left hand blade, which, after being adjusted, was held in position by Dr. Peachy. I then proceeded to adjust the right hand blade, but when introducing my left hand, in order to guide the blade and protect the soft parts, I found it necessary, upon introducing the blade to the point of atresia, to extend the right incision, which I did, and then easily glided the blade over the right parietal bone to its proper position. The blades were then readily locked.

The head was situated obliquely about the centre of the pelvic cavity. Rotation had not taken place. Consequently, the lock of the blades looked to the mother's left groin.

After making slight traction in order to learn whether any portions of the mother's organs were embraced in the instrument, I proceeded to extract, simultaneously with the pains, and downwards and backwards. As soon as the head reached the floor of the pelvis and the occiput appeared under the pubic arch, I gradually elevated the handles of the instrument towards the mother's abdomen, and the forehead and face glided over the perineum. The head was deliverd without even lacerating the fourchette. I then removed the forceps, but found it necessary to extract the shoulders.

Thus the case was terminated, with safety to the mother and child. It is a male child, vigorous and largely above the average size. After separating the child, and waiting some fifteen minutes, I proceeded to deliver the placenta, which was effected without difficulty.

I then requested Dr. Peachy te apply the bandage, and after being satisfied there was no danger of hæmorrhage, left the subsequent management of the case to the two estimable and intelligent gentlemen, Drs, Pendleton and Gardner.

I am under many obligations to my friend, Dr. Peachy, for the efficient assistence he rendered me in the conduct of this interesting case, and also to the gentlemen who were in attendance.— *Virginia Med. Jour.*

